# Salinity‐driven ecology and diversity changes of heterocytous cyanobacteria in Australian freshwater and coastal‐marine microbial mats

**DOI:** 10.1111/1462-2920.16225

**Published:** 2022-10-10

**Authors:** Matthew A. Campbell, Thorsten Bauersachs, Lorenz Schwark, Bernadette C. Proemse, Rolan S. Eberhard, Marco J. L. Coolen, Kliti Grice

**Affiliations:** ^1^ Western Australian Organic and Isotope Geochemistry Centre, School of Earth and Planetary Sciences Curtin University Perth Western Australia Australia; ^2^ Institute of Geosciences, Organic Geochemistry Group Christian‐Albrechts‐University Kiel Germany; ^3^ Institute for Marine and Antarctic Studies University of Tasmania Battery Point Tasmania Australia; ^4^ Natural and Cultural Heritage Division Department of Primary Industries Parks, Water and Environment Hobart Tasmania Australia

## Abstract

N_2_‐fixing heterocytous cyanobacteria are considered to play a minor role in sustaining coastal microbial mat communities developing under normal marine to hypersaline conditions. Here, we investigated microbial mats growing under different salinities from freshwater mats of Giblin River (Tasmania) to metahaline and hypersaline mats of Shark Bay (Western Australia). Analyses of genetic (rRNA and mRNA) and biological markers (heterocyte glycolipids) revealed an unexpectedly large diversity of heterocytous cyanobacteria in all the studied microbial mat communities. It was observed that the taxonomic distribution as well as abundance of cyanobacteria is strongly affected by salinity. Low salinity favoured the presence of heterocytous cyanobacteria in freshwater mats, while mats thriving in higher salinities mainly supported the growth unicellular and filamentous non‐heterocytous genera. However, even though mRNA transcripts derived from heterocytous cyanobacteria were lower in Shark Bay (<6%) microbial mats, functional analyses revealed that these diazotrophs were transcribing a substantial proportion of the genes involved in biofilm formation and nitrogen fixation. Overall, our data reveal an unexpectedly high diversity of heterocytous cyanobacteria (e.g. *Calothrix*, *Scytonema*, *Nodularia*, *Gloeotrichia*, *Stigonema*, *Fischerella* and *Chlorogloeopsis*) that had yet to be described in metahaline and hypersaline microbial mats from Shark Bay and that they play a vital role in sustaining the ecosystem functioning of coastal‐marine microbial mat systems.

## INTRODUCTION

Cyanobacteria likely originated more than 2.5 billion years ago, evolving through periods of dramatic oxygen increases, CO_2_ declines and climatic variations throughout Earth's history (Sánchez‐Baracaldo et al., 2021). Extant cyanobacteria are the most broadly dispersed group of photosynthetic prokaryotes present in virtually every region of the world (Bullerjahn & Post, 2014; W. Tang et al., 2019). They are common in modern marine, freshwater and terrestrial environments (Sánchez‐Baracaldo & Cardona, 2020), and often pioneer microbial colonization of harsh habitats, including drylands, deserts, glaciers and hypersaline ecosystems (Hoffman, 1999; Segawa et al., 2017; Stal, 2012). Within the prokaryotic realm, cyanobacteria are one of the most morphologically diverse groups with species showing simple unicellular to complex filamentous forms (Schirrmeister et al., 2013). The latter have evolved multiple specialized cell types, including heterocytes for biological N_2_ fixation, spore‐like akinetes, and motile hormogonia filaments (Kumar et al., 2010). Nitrogen‐fixing cyanobacteria evolved heterocytes as a response to oxygen exposure, as the enzyme required for nitrogen fixation becomes inactive in the presence of free oxygen (Fay, 1992). This adaption enabled nitrogen fixation to occur in oxic conditions (Boyd & Peters, 2013). Because of this highly sophisticated specialization, heterocytous cyanobacteria are considered as the being best adapted for diazotrophic oxygenic photosynthetic growth under fully oxic conditions (Stal, 1995). Furthermore, they have an important ecological role in many ecosystems, not only as primary producers, but also because of their coexistence with other organisms to which they supply nitrogen. However, they can also have a negative impact on the environment due to their ability to release a range of toxic compounds brought on by sudden environmental shifts (Sciuto & Moro, 2015). Additionally, due to their ancient origin, fossilized molecular markers derived from these organisms have aided in palaeoreconstructions of various environments (Bauersachs et al., 2011). Therefore, gaining further insights into the taxonomic diversity and ecological function of heterocytous cyanobacteria is essential for understanding past, present and future environments.

Microbial mats are typically stratified ecosystems that consist of diverse prokaryotic phyla, including cyanobacteria, anoxygenic photosynthetic bacteria, green sulfur bacteria and purple bacteria, aerobic heterotrophs and anaerobes, such as sulfate‐reducing bacteria, sulfur‐oxidizing bacteria and methanogenic archaea (Prieto‐Barajas et al., 2018). Within these communities, cyanobacteria are of particular importance because they are pioneers in mat development that act as primary producers (Kirk Harris et al., 2013; Ley et al., 2006; Stal, 2012) and atmospheric nitrogen fixers (Bauersachs et al., 2011; Díez et al., 2007), providing bioavailable nitrogen and energy sources to the complex consortium of auto‐ and heterotrophic bacteria. Cyanobacteria are also responsible for the production of extracellular polymeric substances (EPS), which play an important role in the formation of microbial biofilms by providing physical protection and resistance to desiccation (Dupraz et al., 2009). Among the most dominant cyanobacteria in freshwater and coastal‐marine microbial mats are the cosmopolitan filamentous genera *Microcoleus*, *Lyngbya* and *Oscillatoria* and the coccoid forms *Synechocystis* and *Synechococcus* (Paerl et al., 2000). N_2_‐fixing heterocytous cyanobacteria, including the genera *Calothrix*, *Nodularia* and *Scytonema*, have also been reported from some intertidal coastal‐marine (Bauersachs et al., 2011; Bolhuis & Stal, 2011) and hypersaline microbial mats (Javor & Castenholz, 1981) but due to the low abundance of heterocytous species, it is commonly believed that they play a minor role in the functioning of microbial ecosystems developing under high salinities.

Shark Bay, Western Australia, contains some of the most extensive microbial mat communities adapted to elevated salinities in the world (Jahnert & Collins, 2011), however little is known about the diversity and functional roles of heterocytous cyanobacteria within these communities. Using a combination of metatranscriptomics and lipidomics, this study examined the taxonomic diversity and ecological functioning of cyanobacteria, with a focus on heterocytous species, occurring in microbial mats from metahaline (45–65) and hypersaline (65–70) environments in Shark Bay. These data were compared to microbial mats from Giblin River, Tasmania, to provide comprehensive insights on diversity and activity changes of heterocytous cyanobacteria between environments of different salinities. Our data indicate that Shark Bay microbial mats harbour communities of heterocytous cyanobacteria taxonomically more diverse and active than previously anticipated and that they play key roles in biogeochemical processes (including N_2_ fixation and biofilm formation). Thus, they are crucial for the functioning of microbial mat systems not only under freshwater conditions but also under elevated salinities.

## MATERIALS AND METHODS

### Site descriptions

**FIGURE 1:**
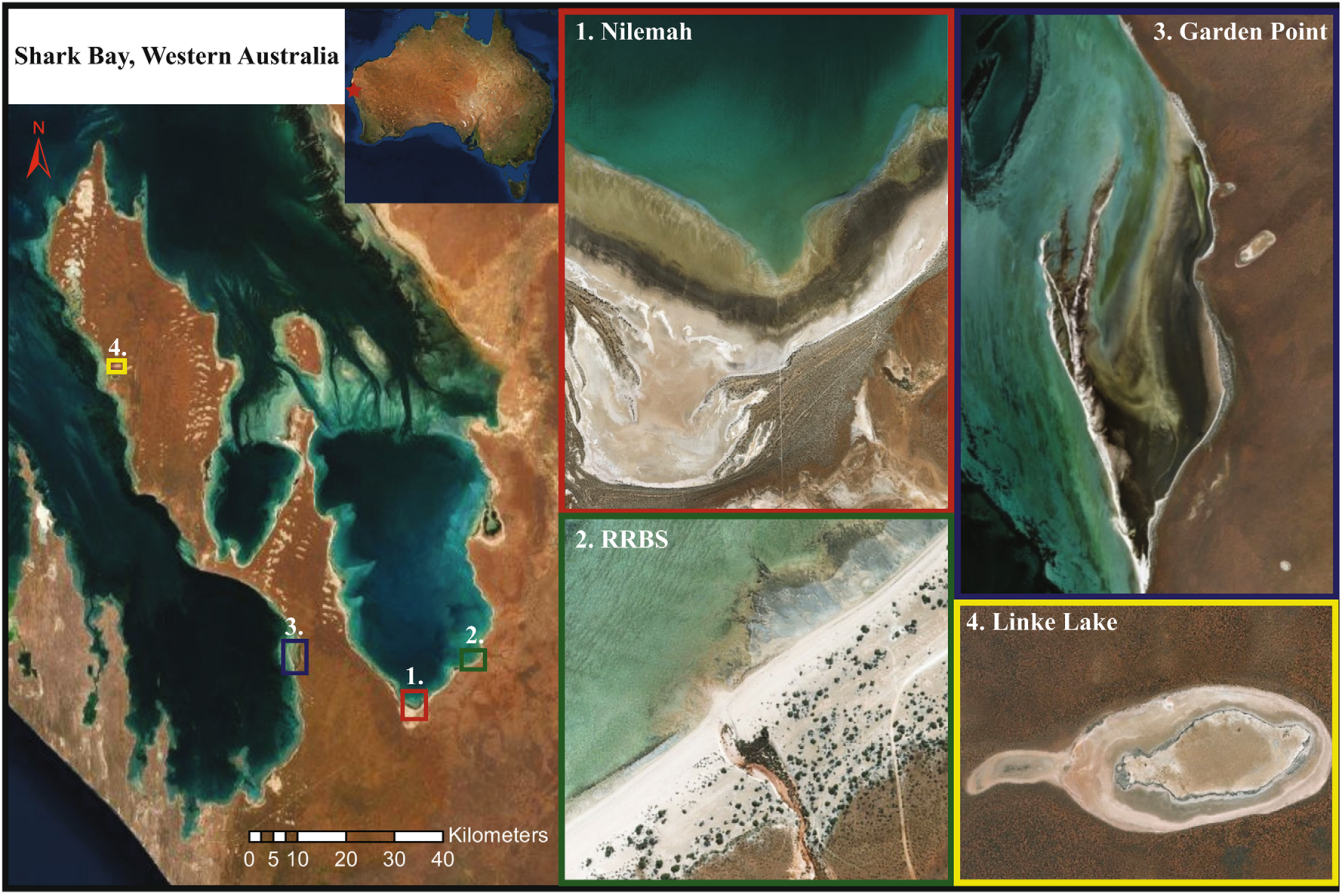
Satellite images of the Shark Bay world heritage area: (1) Nilemah, (2) RRBS, (3) Garden Point and (4) Linke Lake

Microbial mats were sampled from four locations in Shark Bay, Western Australia: Garden Point, Nilemah, Ron's Running Beach South (RRBS) and Linke Lake (Figure 1). Garden Point is a small re‐entrant of about 11 km^2^, located in the eastern area of Henri Freycinet embayment (26°36′ S, 113°88′ E). This location is characterized by metahaline conditions (salinity ranging between 40 and 56 ppt) with pH ranges between 7.8 and 8.7 (Jahnert & Collins, 2013). Nilemah is an intertidal flat located in the southern area of Hamelin Pool (26°27′ S, 114°05′ E), characterized by a littoral gradient that varies from 20 to 150 cm km^−1^, restricting tidal influxes, and laterally well‐defined tidal zonation (Campbell et al., 2020; Jahnert & Collins, 2013). The site referred to as ‘RRBS’ is an intertidal terrace located in a south‐eastern region of Hamelin Pool (26°37′ S, 114°20′ E). RRBS was significantly impacted by tropical cyclone (TC) activity in 2015 and lies at the southern end of a bight (sensu Logan & Cebulski, 1970), north of Flint Cliff, at the mouth of an outwash drainage channel that flows episodically into an intertidal terrace during rare high rainfall events such as during TC Olwyn (Campbell et al., 2021; Morris et al., 2020). Restricted circulation in conjunction with high rates of evaporation and limited rainfall lead to the water in Hamelin Pool to be hypersaline (60–70 ppt) with pH ranges between 7.5 and 8.0 (Jahnert & Collins, 2013; Suosaari et al., 2016). Linke Lake is a gypsum lake located north of Denham in the Francois Peron National Park (25°88′ S, 113°55′ E). The lake is oval in shape, 1 km wide and contains a central, raised platform ringed by a moat‐like depression containing hypersaline evaporative ponds (salinity >80 [beyond probe detection], evaporative ponds are typically between 200 and 300 ppt) with a pH of 7.8 (Bufarale & Collins, 2015).

**FIGURE 2:**
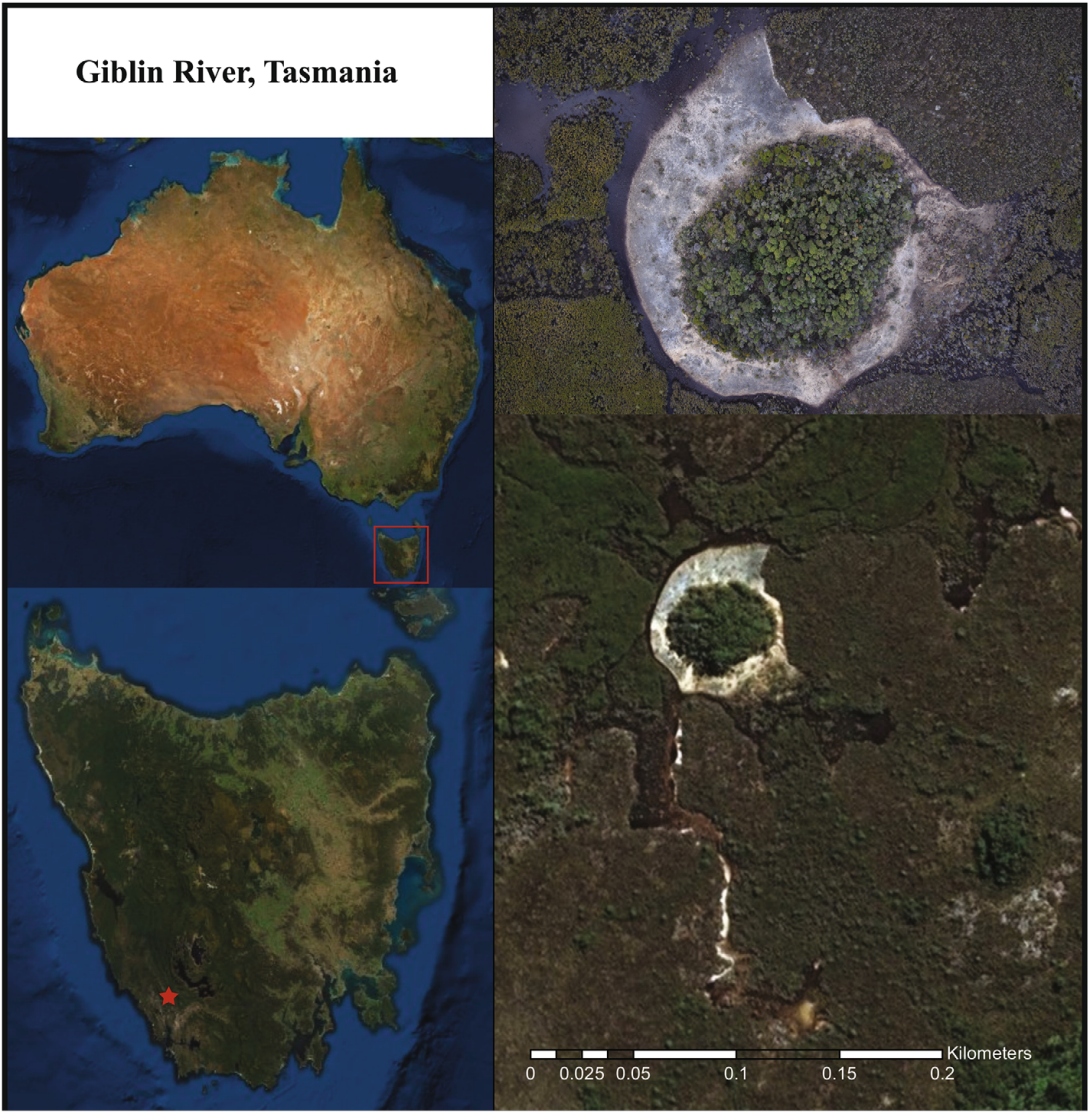
Satellite images of the Giblin River UNESCO‐listed Tasmanian Wilderness World Heritage Area

Freshwater microbial mats, growing on the wetted surface of tufa barriers and described as ‘stromatolitic smooth mats’ (Proemse et al., 2017), were discovered in the Giblin River catchment located in south‐west Tasmania, Australia (42°56′ S, 145°45′ E) in 2015. This site was revisited in August 2016 to collect microbial mats from gravelly flats surrounding spring mounds for this study. Giblin River lies within the United Nations Educational, Scientific and Cultural Organization (UNESCO) listed Tasmanian Wilderness World Heritage Area (Figure 2). The Giblin River catchment is a low relief limestone basin mantled by extensive blanket bog peat soils. Groundwater discharge at springs on the floor of the basin has created peat‐bound karstic wetlands (PKWs), a form of groundwater dependent ecosystem conditioned by the steep pH gradient between the alkaline spring and the surrounding acidic peat. The Giblin River PKWs are regionally distinctive due to the presence of prominent spring mounds and carbonate deposition as tufa. These mounds are up to 60 m in diameter with densely vegetated marshy tops, rising to 1.8 m above surrounding wetlands, which are typically bare gravelly flats. The freshwater karstic spring mounds (salinity of 0.05–0.25 ppt) are mildly alkaline with pH ranges between 7.0 and 7.9 (Proemse et al., 2017).

### Sample descriptions

**FIGURE 3:**
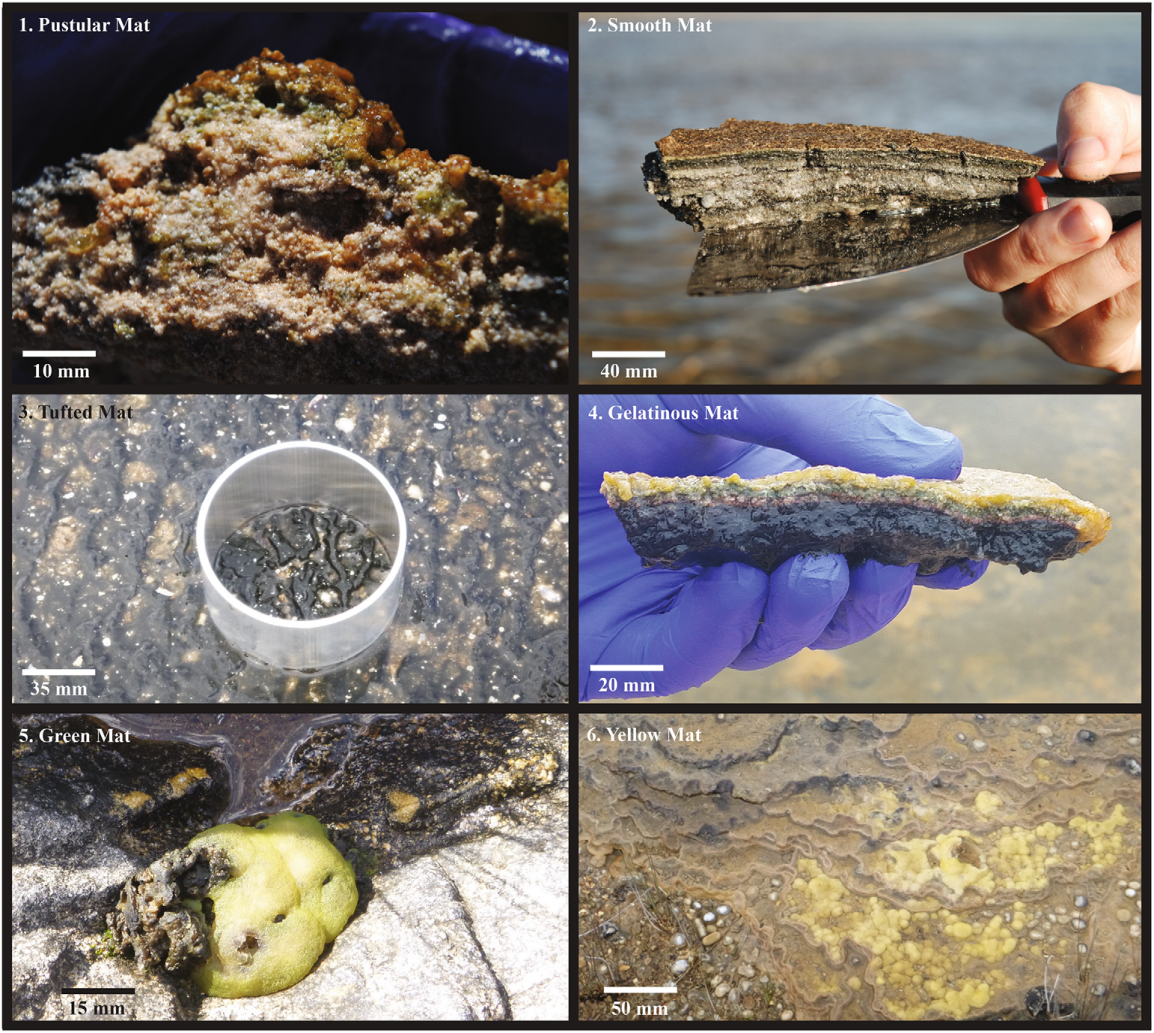
Field images of (1) pustular and (2) smooth mats from Nilemah; (3) tufted mat from Garden Point; (4) gelatinous (birrida) mat from Linke Lake; (5) green and (6) yellow mats from Giblin River. Photography credits to Alex Sessions (1 and 2) and Yalimay Jiménez (4)

Pustular mats (PMs) were found in Nilemah and Garden Point; these mats had a dark pigmented surface layer with a pustular or crenulated surface (Figure 3 (1)). The surface layer of these mats had a thickness of 1–8 mm through an individual pustule. No clear lamination was visible beneath the surface layer, although patches of green (cyanobacteria), pink (purple sulfur bacteria) and black (sulfate reducers) colouration indicates that micro‐compartments of microorganisms occur within the medium‐grain irregular fenestral fabric of this mat (Logan et al., 1974). Smooth mats (SM) were present in Nilemah, RRBS and Garden Point; these mats had a light brown visible band of cyanobacteria present 1–2 mm beneath the surface (Figure 3 (2)). This mat type had distinct lamination of subsurface sediments with purple/pink (2–6 mm deep) and black (6–10 mm deep) zones visible in the fine to medium laminoid fenestral fabric (Logan et al., 1974; Pagès et al., 2014; Plet et al., 2018). Tufted mats (TMs) grew in RRBS and Garden Point (1–5 mm thick) and consisted of a greenish brown to black colour. They were characterized by corrugated surfaces of sharp‐crested ridges and low, broad depressions (Figure 3 (3)). The ridges are formed by vertically oriented tufts composed of large filaments of *Lyngbya aestuarii* (Suosaari et al., 2018). The sample described as ‘ooze over sand’ (OVS) was collected from RRBS, initially described as a thin mucilaginous sheet deposited as floc/ooze on a newly formed sand layer that occurred after TC Owlyn in 2015; a year later this material was found to be transitioning into a microbial mat and was sampled for this study. The gelatinous (birrida) microbial mat collected from Linke Lake was ~12 mm thick with a smooth slimy yellow‐brown surface layer (1–4 mm deep), covering laminated subsurface layers with green (4–8 mm deep), purple/pink (8–11 mm deep) and black (>11 mm deep) zones (Figure 3 (4)). Green (GM) and yellow mats (YM) occurred at Giblin River (Figure 3 (5, 6)). These mats were ~20–30 mm thick with a smooth surface layer and contained either greenish or yellowish sheet‐like structures with calcite laminations (Proemse et al., 2017).

### Field sampling

Microbial mats from Shark Bay were sampled using aluminium push cores (20 cm × 10 cm), whereas Giblin River mats were collected with sterilized spatulas and wrapped in aluminium foil afterwards. Both the aluminium foil and push cores were annealed at 550°C prior to field sampling. All mats were sampled during daylight hours with additional sampling of PM and SM during the night from the Nilemah tidal flat. The top 20 mm of the ‘active’ mats were immediately subsampled in triplicate for transcriptomic analysis using UV sterilized open‐ended single‐use 5 ml syringes and then placed into sterile tubes containing RNAlater® (Thermo Fischer Scientific, MA, USA). Cores and mat samples along with the samples for transcriptomic analysis were frozen at −20°C for transport in the field. Samples for biomarker analysis were kept frozen at −20°C, whereas the transcriptomic samples were stored at −80°C in the laboratory until nucleic acid extraction. Physiochemical parameters of the surrounding water at each site (i.e. pH, salinity and temperature) were recorded using a multi‐probe system (HI 9829 Multiparameter, Hanna® Instruments [Shark Bay] and Hach multimeter [Giblin River]), refractometer and pH meter. A detailed summary of the mat types collected with sampling dates and times, and field measurements are given in Table S1.

### RNA isolation, library preparation and sequencing

The RNeasy PowerSoil Total RNA Kit (Qiagen, Hilden, Germany) was used to extract total RNA (in triplicate) from 50 to 100 mg of sample per reaction. An additional DNase treatment with the Turbo DNA‐free Kit (Qiagen) was used to remove residual DNA and the DNA‐free RNA extracts were purified using the MEGAclear kit (Thermo Fischer Scientific, MA, USA). RNA concentrations were measured with a NanoDrop 3300 (Thermo Fisher Scientific), using the Quant‐iT™ RiboGreen™ RNA Assay Kit (Thermo Fisher Scientific). SYBR green‐based quantitative PCR targeted bacterial 16S rDNA (V4 region) was applied to test if the DNase‐treated RNA samples were completely free of DNA. Equimolar amounts of the triplicate DNA‐free RNA samples were pooled for the synthesis of cDNA using the Ovation RNAseq System V2 kit and subsequent library preparation using the Ovation Ultralow Library System V2 kit (NuGEN Technologies, CA, USA) following the manufacturer's protocol. cDNA libraries were analysed for quality and quantity with a 2100 bioanalyzer (Agilent, CA, USA). Barcoded libraries were diluted in 10 mM Tris–HCl at pH 8.5 (25 μl) and sent to the Australian Genomic Research Facility (AGRF). At AGRF, the Illumina HiSeq 2500 platform was used to generate 2 × 125‐bp pair‐end sequencing reads. The HiSeq Control Software (HCS) v2.2.68 and Real Time Analysis (RTA) v1.18.66.3 software performed real‐time image analysis and base calling on the HiSeq instrument computer. The AGRF Illumina bcl2fastq 2.20.0.422 pipeline was used to generate the sequence data.

### Bioinformatics

Taxonomic analysis of the microbiota was based on assembled 16S rRNA transcripts, followed by functional and taxonomic annotation of transcripts derived from the mRNA fraction. Raw paired‐end Illumina sequenced reads were trimmed with BBDuk. BBDuk was ran twice; (1) to trim adapters with parameters ktrim = r, *k* = 21, mink = 11, hdist = 2, tpe, tbo and (2) for quality trimming with parameters qtrim = r, trimq = 25, maq = 25, minlen = 50, *k* = 31, qhdist = 1 (Bushnell, 2014). Bowtie2 was used to map rRNA and PhiX sequences, then Samtools was used to separate and convert sequences into mapped and unmapped fastq files (Langmead & Salzberg, 2012; H. Li et al., 2009). Fastq‐pair was used to match the paired‐end mapped and unmapped reads, to ensure that all reads were paired and to separate out singletons (Edwards & Edwards, 2019). For taxonomic analysis of the mapped reads (rRNA sequences), the paired end mapped reads were analysed using the Phyloflash pipeline (v.3.3). Reads encoding 16S and 18S rRNA gene sequences were aligned to sequences from the SILVA SSU132 NR99 database with BBmap, with minimum identity of 96% and read limit of <5,000,000 (Gruber‐Vodicka et al., 2020). Taxonomic classification for heterocytous cyanobacteria were based on the National Center for Biotechnology Information (NCBI) taxonomy index (Federhen, 2012). For functional analysis of unmapped reads (mRNA sequences), the unmapped paired‐end reads were checked again for presence of rRNA and phiX sequences using Bowtie2 then assembled into transcripts using rnaSPAdes (v.3.13.0) with the default *k*‐mer size of *k* = 55 (Bushmanova et al., 2019). To decipher the functionality of heterocytous cyanobacteria, protein sequences predicted with Prodigal (v.2.6.1) were annotated using eggNOG‐mapper (v.2.0.1) in ‘diamond’ run mode with the eggNOG database (v.5) (Huerta‐Cepas et al., 2019). Transcription of genes involved in photosynthesis (KEGG map00195), CO_2_ fixation (KEGG map00710/20), biofilm (EPS) formation (KEGG map02024/5/6 and map05111) and nitrogen fixation (KEGG map00910) were assessed to determine if heterocytous cyanobacteria played a critical role for the functioning of the microbial ecosystems. Day and night samples from Nilemah were further assessed for nitrogen fixation gene transcripts. Illumina HiSeq 2500 pair‐end sequencing read output with percentage summaries of trimmed and aligned sequences, and number of Phyloflash and eggNOG‐mapper annotations are provided in Table S2.

### Lipid extraction and analysis

Lipid extraction from the top ~10 mm of the core or biomass containing the ‘living’ mat section was performed using a modified Bligh and Dyer procedure (Allen et al., 2010; Bligh & Dyer, 1959). The organic layer was transferred to a collection tube containing freshly activated copper turnings and stirred at room temperature for 72 h to remove elemental sulfur. The sulfur‐free Bligh and Dyer extracts (BDEs) were filtered over anhydrous magnesium sulfate (MgSO_4_) and dried gently under a nitrogen purge. Activated silica gel column chromatography (5.5 cm × 0.5 cm i.d.) was performed to separate BDEs (≥10 mg) into neutral and polar lipid fractions. The neutral lipid fractions were eluted with DCM (7 ml) and the polar fractions with MeOH (14 ml) (Heinzelmann et al., 2014). Polar fractions, containing heterocyte glycolipids (HGs), were analysed using a Waters Alliance 2690 HPLC system coupled to a Micromass Quattro LC triple quadrupole mass spectrometer, following previously established analytical protocols (Bauersachs et al., 2015). HGs were detected in MS/MS mode and identified using transitions specified earlier (Bale et al., 2018; Bauersachs et al., 2013; Bauersachs, Hopmans, et al., 2009; Schouten et al., 2013; Wörmer et al., 2012) and by comparison of HG retention times of components found in the microbial mats with those found in axenic cyanobacterial cultures. Long chain HGs (≥30 carbon atoms) with pentose headgroups found previously in marine cyanobionts, for which no cell material or standards are commercially available, were identified based on comparison with mass spectral characteristics reported in the literature (Schouten et al., 2013). HGs were quantified by integrating peak areas using the QuanLynx application software.

### Biostatistical analysis

A variety of R packages (R Core Team, 2017) were used to analyse SSU rRNA, HG and transcriptomic results. Analysis of similarity (ANOSIM) using the vegan package with 999 permutations was conducted to determine whether the dissimilarity of HGs and cyanobacterial proportion of the microbial communities between different locations was significant (Dixon, 2003). Dotplots of the normalized transcript, SSU rRNA and HG classifications were completed using Reshape2 with the melt function then plotted using ggplot2 showing the relative abundance as a percentage (Hadley, 2015). Principle component analysis (PCA) of the normalized HG and SSU rRNA classifications used Ecodist (dissimilarity‐based functions for ecological analysis), and pvclust (hierarchical clustering with *p*‐values via multiscale bootstrap resampling) using ward clustering and Bray‐Curtis distance metrics at a thousand replicates (Goslee & Urban, 2007; Suzuki & Shimodaira, 2006). *k*‐means cluster analysis using the ggplot2 and ggfortify packages (Y. Tang et al., 2016), and multilevel pattern analysis using the indicspecies package (De Cáceres & Legendre, 2011) were applied to further assess groupings of sampling caused by particular HGs or cyanobacterial genera. To investigate correlations between cyanobacterial and HG distributions, a regularized canonical correlation analysis (rCCA) was performed using the package mixOmics (Rohart et al., 2017). To evaluate gene transcription in heterocytous cyanobacteria, differential analysis of transcribed genes was calculated from the variance stabilizing transformation of KO (KEGG Orthology) count data using the DESeq2 package and visualized using pheatmap (Love et al., 2014). Transcribed gene counts lower than 10 were excluded from statistical analyses.

## RESULTS

### Microbial communities and cyanobacterial distribution

Based on rRNA transcripts, metahaline and hypersaline microbial mats were predominately dominated by Proteobacteria (α‐ [12.4 ± 5.2%], δ‐ [17.1 ± 8.0%], γ‐ [7.7 ± 4.2%] classes), as well as Bacteroidetes (14.5 ± 6.1%), Planctomycetes (12.1 ± 7.3%), Cyanobacteria (11.9 ± 8.1%), Chloroflexi (7.1 ± 4.3%), and Spirochaetes (4.3 ± 4.0%) (Figure S1). In contrast, freshwater microbial mats were dominated by Proteobacteria, with the α‐ (40.7 ± 9.9%) class being predominant in green mats, whereas δ‐ (58.6%) and γ‐ (21.9%) classes were most dominant in the YM. Other major bacterial groups in these mats included Planctomycetes (12.2 ± 6.5%), Cyanobacteria (12.0 ± 6.0%), Bacteroidetes (2.9 ± 2.2%), Chloroflexi (2.2 ± 0.9%), and Armatimonadetes (1.3 ± 0.9%).

The recovery of rRNA and mRNA transcripts provided the opportunity to use two different approaches to assess the contributions of living and metabolically active cyanobacteria to the overall mat community. Cyanobacterial rRNA transcripts accounted for an average 13.2 ± 9.3% of total reads in hypersaline mats, 8.6 ± 3.5% in metahaline mats and 11.8 ± 5.7% in freshwater mats (Table S3). In contrast, mRNA transcripts accounted for a larger proportion with an average 29.1 ± 9.6% of total reads in hypersaline mats, 21.0 ± 6.5% in metahaline mats and 22.0 ± 13.3% in freshwater mats (Table S4). 16S rRNA transcripts of non‐heterocytous cyanobacteria were on average 9 times more abundant in hypersaline mats and 21 times in metahaline mats than 16S rRNA transcripts of heterocytous cyanobacteria, whereas in freshwater mats, heterocytous cyanobacterial 16S rRNA was on average twice as abundant as non‐heterocytous cyanobacterial 16S rRNA. In the cyanobacterial mRNA transcripts, heterocytous cyanobacteria accounted for a larger proportion of the reads with non‐heterocytous cyanobacteria being on average three times more abundant in hypersaline mats and two times more abundant in metahaline mats. In the freshwater mat GR GM1 2016, heterocytous cyanobacteria were four times more abundant than the non‐heterocytous cyanobacteria. However, the other two freshwater mats were found to have similar abundances of heterocytous and non‐heterocystous cyanobacteria. Oscillatoriales and Synechococcales were the prominent non‐heterocytous cyanobacteria in 16S rRNA transcripts from mats occurring in saline environments. However, Synechococcales was not annotated in the cyanobacterial mRNA transcripts. Instead, Chroococcales and Oscillatoriales were the most annotated functional genes expressing non‐heterocytous cyanobacteria. Heterocytous cyanobacteria of the orders Nostocales and Stigonematales accounted for <1% 16S rRNA transcripts in both the hypersaline and metahaline mats except for Nil PM1 2016 having a slightly higher relative abundance of Nostocales (2.3%). In the Giblin River freshwater mats, heterocytous cyanobacteria contributed 3.1%–5.9% of the total 16S rRNA transcripts (Table S3). Whereas Nostocales and Stigonematales collectively accounted for 2.5%–9.5% of the cyanobacterial mRNA transcripts in both the hypersaline and metahaline mats and 1.1%–25.6% in the freshwater mats (Table S4).

### Functional role of cyanobacteria

Heterocytous cyanobacteria accounted for 9%–16% of the photosynthesis gene transcripts (e.g. *psa*ABCD; *rbcL*) in the meta‐ to hypersaline mats. In the freshwater mats, they were highly variable, ranging from 5% to 66% (Table S5). Overall, large proportions of the photosynthesis‐related gene transcripts were attributed to the non‐heterocytous cyanobacteria Chroococcales (10%–70%) and Oscillatoriales (7%–15%). Heterocytous cyanobacteria belonging to the Nostocales accounted for 3%–15% of the CO_2_ fixation gene transcripts (e.g. PRK, prkB [phosphoribulokinase]) in the marine microbial mats. In the freshwater mats, their contribution was more variable and ranged from 3% to 44% (Table S6). Overall, most CO_2_ fixation gene transcripts were associated with the non‐heterocytous cyanobacteria Chroococcales (22%–58%) and Oscillatoriales (4%–44%).

Both heterocytous and non‐heterocytous cyanobacteria also accounted for a large proportion of the gene transcripts involved in biofilm (EPS) formation (e.g. wza, gfcE [polysaccharide biosynthesis/export protein]) with Nostocales accounting for 7%–35% and Chroococcales for 0%–31% in meta‐ to hypersaline microbial mats (Table S7). In freshwater mats, Nostocales accounted with 2%–42% (average 28 ± 22%) for a large fraction of the gene transcripts involved in EPS formation. Other bacterial phyla (such as Actinobacteria, Bacteroidetes, Chloroflexi, Firmicutes and Proteobacteria) together contributed with 16%–38% to the transcripts involved in EPS formation in PMs, as well as freshwater mats.

**FIGURE 4:**
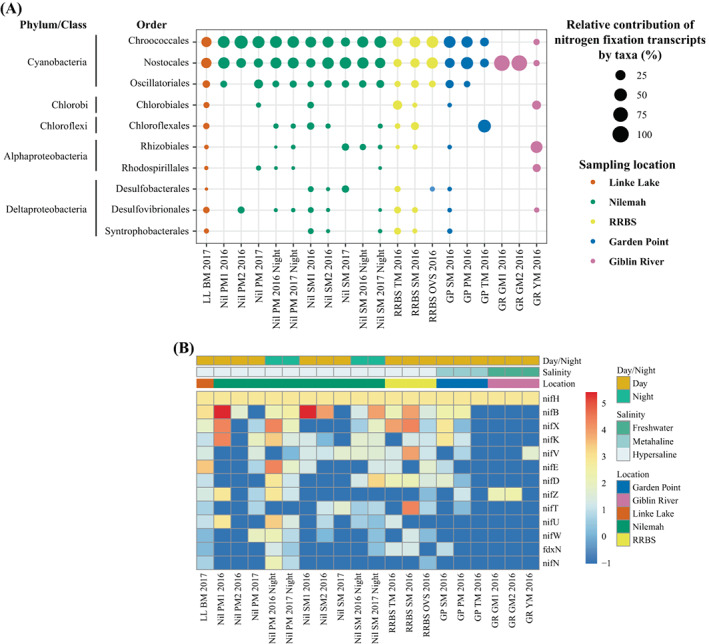
Metatranscriptomic profiling of nitrogen fixation in microbial mats. (A) Relative transcript abundances from taxa transcribing genes involved in nitrogen fixation. (B) Heatmap showing 13 nitrogen fixation genes abundantly transcribed (>10) in Nostocales transcriptomes. The *nif* genes encode enzymes involved in the fixation of atmospheric nitrogen and *fdx* genes encode ferredoxin‐like proteins in the *nif* region. Differential analysis of the transcribed genes was calculated from the variance stabilizing transformation of KO count data. The gradient from red to blue indicates gene abundance across samples with red representing genes that are highly transcribed and blue indicating genes that have lower relative transcription.

In saline mats, cyanobacteria accounted for the largest proportion of the gene transcripts involved in nitrogen fixation with Chroococcales accounting for 20%–64%, Oscillatoriales for 6%–22% and Nostocales for 16%–52% (Figure 4A; Table S8). Only cyanobacteria of the order Nostocales were found to be transcribing nitrogen fixation genes in the green freshwater mats (100%). However, they only accounted for 7% in the yellow freshwater mat, in which α‐Proteobacteria (Rhizobiales) and green sulfur bacteria (Chlorobiales) were the largest contributors of fixed N. Comparison of mats sampled during the day and night from Nilemah indicated no relative change in transcription by cyanobacterial groups. Thirteen nitrogen‐fixing genes were found to be transcribed by Nostocales with the nitrogenase iron protein (*nif*H) being abundantly transcribed in all the mats studied (Figure 4B). Transcription of nitrogen‐fixing genes was considerably higher and diverse in Nostocales occurring in saline environments. Abundantly transcribed nitrogen‐fixing genes by Nostocales in saline mats included nitrogenase *nif*BDEHKNTUVWXZ genes and fdxN (ferredoxin‐like protein in the *nif* region). Nostocales occurring in the freshwater mats were found to only transcribe *nif*HVZ genes.

### Distribution of heterocytous cyanobacteria

**FIGURE 5:**
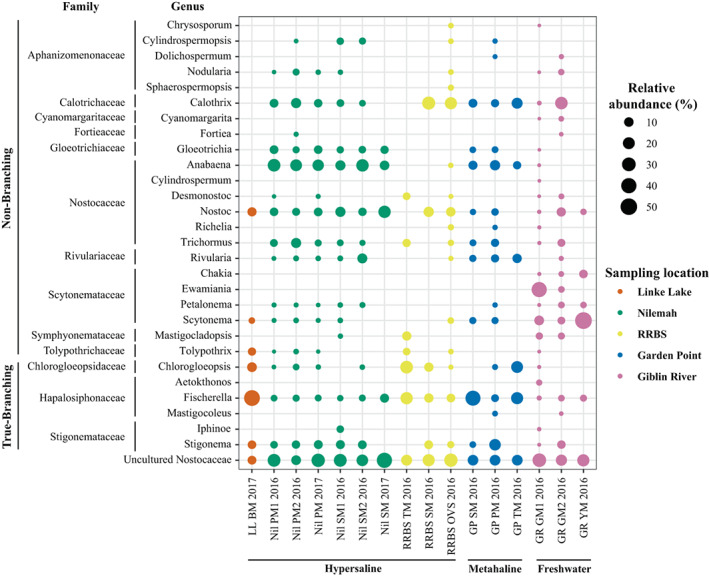
Dot plot based on 16S rRNA transcript analysis displaying the composition and relative abundance of the non‐branching (order Nostocales) and true‐branching (order Stigonematales) heterocytous cyanobacteria in microbial mats from Shark Bay, Western Australia (Linke Lake [LL], Nilemah [Nil], RRBS and Garden Point [GP]) and Tasmania (Giblin River [GR]). BM, birrida mat (gelatinous mat); GM, green mat; OVS, ooze over sand; PM, pustular mat; SM, smooth mat; TM, tufted mat; YM, yellow mat

**FIGURE 6:**
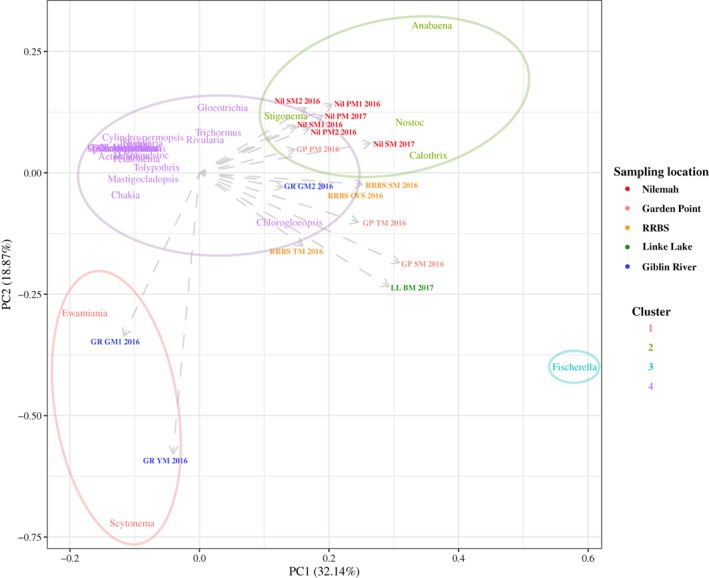
Principle component analysis (PCA) plot constructed from *k*‐means cluster analysis of active heterocytous genera within microbial mats from Shark Bay, Western Australia (Linke Lake [LL], Nilemah [Nil], RRBS, Garden Point [GP]) and Tasmania (Giblin River [GR]). BM, birrida mat (gelatinous mat); GM, green mat; OVS, ooze over sand; PM, pustular mat; SM, smooth mat; TM, tufted mat; YM, yellow mat

Based on 16S rRNA transcript analysis, heterocytous cyanobacteria significantly differed between sampling sites (ANOSIM, *R* = 0.61; *p* = 0.0001), consisting of 10 non‐branching nostocalean families (*Aphanizomenonaceae*, *Calotrichaceae*, *Cyanomargaritaceae*, *Fortieaceae*, *Gleotrichiaceae*, *Nostocaceae*, *Rivulariaceae*, *Scytonemataceae*, *Symphyonemataceaea* and *Tolypothrichaceae*), and three true‐branching stigonematalean families (*Chlorogloeopsidaceae*, *Hapalosiphonaceae* and *Stigonemataceae*) (Figure 5). *k*‐means cluster analysis demonstrated that microbial mats from Nilemah had greater inputs from the genera *Anabaena*, *Stigonema*, *Nostoc*, *Rivularia* and *Calothrix* (Cluster 2); RRBS and Garden Point from *Calothrix*, *Fischerella* and *Chlorogloeopsis* (Clusters 3 and 4); Linke Lake from *Fischerella* (Cluster 3); and Giblin River from *Scytonema* and *Ewamiania* (Cluster 1; Figure 6; Table S9). PCA of the data set revealed that microbial mats typically grouped within their sampling locations, although especially low variance was observed between Nilemah and Garden Point mats (Figure S2). Furthermore, TMs from RRBS and Garden Point grouped together, as well as the OVS from RRBS and the gelatinous (birrida) mat from Linke Lake grouping together with the Giblin River microbial mats.

### Distribution of HGs

**FIGURE 7:**
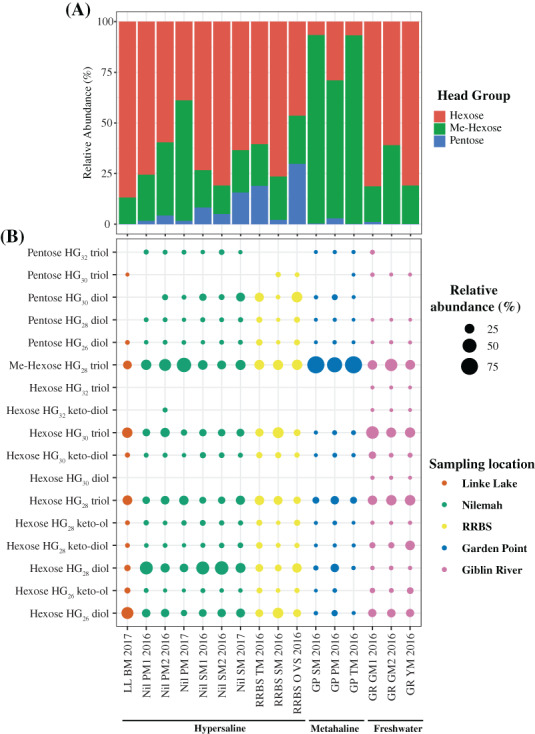
(A) Bar chart displaying the summed composition and relative abundance of hexose, methyl‐hexose and pentose headgroups in glycolipids. (B) Dot plot displaying the composition and relative abundance of heterocyte glycolipids (HGs) in microbial mats from Shark Bay, Western Australia (Linke Lake [LL], Nilemah [Nil], RRBS and Garden Point [GP]) and Tasmania (Giblin River [GR]). BM, birrida mat (gelatinous mat); GM, green mat; OVS, ooze over sand; PM, pustular mat; SM, smooth mat; TM, tufted mat; YM, yellow mat

HGs have not been reported in any organism other than heterocytous cyanobacteria and their distribution in these diazotrophs suggests a chemotaxonomic relevance that might allow distinguishing between species of different genera and families, as well as representing excellent tracers for nitrogen fixation in modern and ancient environments (Bauersachs et al., 2011). Seventeen HG structures were found in the Shark Bay and Giblin River microbial mats (Figure 7). ANOSIM showed that HG distribution patterns differed significantly between sampling locations (*R* = 0.92; *p* = 0.0001). For example, multilevel pattern analysis indicated that pentose HG_30_ diol occurred significantly more often in microbial mats from Nilemah, Garden Point and RRBS (*p* = 0.047) (Table S10). Freshwater microbial mats from Giblin River exclusively contained hexose HG_30_ diol and hexose HG_32_ triol (*p* = 0.009). In general, freshwater microbial mats were dominated by HGs with hexose headgroups that on average constituted 74.4 ± 9.5% of the total HG content (Figure 7). In contrast, in hypersaline microbial mats, these HGs were more variable averaging 65.6 ± 22.1%. Metahaline microbial mats from Garden Point received the lowest contribution from HGs with hexose headgroup with on average 14.1 ± 10.5%. Hypersaline microbial mats showed with 8.5 ± 8.9% the highest proportion of HGs with pentose headgroups. These HGs made only small contributions in metahaline and freshwater microbial mats ranging from 1.1 ± 1.3% to 0.4 ± 0.5%, respectively. Me‐hexose HG_28_ triol was on average the most dominant HG across all the mats studied (36.1 ± 26.3%), especially within metahaline mats from Garden Point, in the latter making up 84.7 ± 11.8% of all HGs. In freshwater and hypersaline mats, Me‐hexose HG_28_ triol occurred in almost equal average abundances of 25.2 ± 9.7% and 24.8 ± 23.2%, respectively.

**FIGURE 8:**
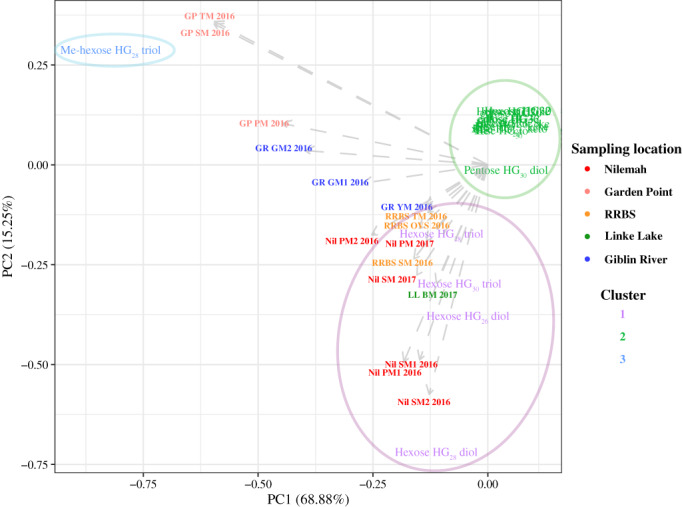
Principle component analysis (PCA) plot constructed from *k*‐means cluster analysis of heterocyte glycolipids (HGs) within microbial mats and microbially derived materials from Shark Bay, Western Australia (Linke Lake [LL], Nilemah [Nil], RRBS, Garden Point [GP]) and Tasmania (Giblin River [GR]). BM, birrida mat (gelatinous mat); GM, green mat; OVS, ooze over sand; PM, pustular mat; SM, smooth mat; TM, tufted mat; YM, yellow mat

*k*‐means cluster analysis confirms that microbial mats from metahaline locations of Garden Point had greater inputs from Me‐hexose HG_28_ triol; microbial mats from the hypersaline locations of Nilemah, RRBS, Linke Lake had greater inputs from hexose HG_26_ diol, hexose HG_28_ diol, hexose HG_28_ triol and hexose HG_30_ triol. Freshwater microbial mats from Giblin River clustered in between the mats from metahaline and hypersaline localities showing a minor preference to the Me‐hexose HG_28_ triol. PCA of HG distributions revealed that microbial mats generally grouped within their sampling locations (Figure 8).

### Lipidomic and taxonomic integration

rCCA revealed that HGs were positively correlated with the community of heterocytous cyanobacteria (Figure S4). For example, a strong positive relationship with hexose HG_28_ diol was observed for *Gloeotrichia* and *Anabaena*. Positive relationships of hexose HG_28_ diol were also observed with *Cylindrospermopsis*, *Nostoc*, *Trichormus* and *Stigonema*. A strong positive relationship between pentose HG_30_ triol, hexose HG_30_ triol, hexose HG_30_ keto‐diol, hexose HG_30_ diol and hexose HG_32_ triol was observed for *Ewaniana*, *Cylindrospermum*, *Aekothonos* and *Mastigocladopsis*. *Chakia* and *Scytonema* showed positive relationships with hexose HG_30_ triol and hexose HG_28_ triol. Hexose HG_28_ triol also had positive relationships with *Ewaniana*, *Cylindrospermum*, *Aekothonos* and *Mastigocladopsis*. A positive relationship with Me‐hexose HG_28_ triol was observed for *Tolypothrix* and to a lesser extent *Calothrix*, *Rivularia* and *Stigonema*.

## DISCUSSION

### Distribution of biologically active heterocytous cyanobacteria in microbial mats

Salinity has been considered to largely exclude heterocytous cyanobacteria from normal marine and hypersaline environments (Oren, 2015). Along the salinity gradient investigated here, low salinity indeed favoured the presence and abundance of heterocytous cyanobacteria. They were most prominent in freshwater mats, while mats occurring in higher salinities were dominated mainly by the presence of non‐heterocytous genera, including Oscillatoriales and Synechococcales. This corroborates with phylogenetic studies on the influence of salinity on cyanobacterial communities (Kirkwood et al., 2008; Oren, 2015). Previous 16S rRNA gene profiling (Goh et al., 2009) and microscopy (Jahnert & Collins, 2013) of Shark Bay microbial mats have detected unicellular and filamentous cyanobacteria of the orders Chroococcales, Pleurocapsales and Oscillatoriales (i.e. *Lyngbya aestuarii* in TMs). Heterocytous cyanobacteria, however, were not detected during microscopic examination and universal bacterial 16S rRNA gene analysis (Allen et al., 2009) in initial studies of Shark Bay microbial mats. More recent metagenomic and amplicon‐focused studies of Shark Bay microbial ecosystems have identified several members of the Nostocales, including *Dichothrix* and *Cylindrospermum* (Babilonia et al., 2018; Garby et al., 2013).

The use of high‐throughput sequencing, newly developed bioinformatics packages and an updated taxonomic database has allowed for enhanced phylogenetic resolution of heterocytous cyanobacteria in both freshwater and elevated salinity environments. 16S rRNA transcripts revealed a high diversity of active non‐branching (e.g. *Anabaena*, *Calothrix*, *Scytonema*, *Nodularia*, *Gloeotrichia* and *Nostoc*) and true‐branching genera (e.g. *Stigonema*, *Fischerella* and *Chlorogloeopsis*) in both the metahaline and hypersaline microbial mats (Figure 5). These genera have previously been found to occur in a range of saline environments (Ali & Sandhu, 1972; Hindák, 2008; Paerl et al., 2000; Pfeffer & Brown, 2016; Oren, 2015; Roney et al., 2009; Sheridan, 1992; Srivastava et al., 2009); however, there are only limited reports of these cyanobacteria in Shark Bay. For example, *Anabaena* has been identified via non‐ribosomal peptide synthetases present in stromatolites (Burns et al., 2005) and from a culture‐based study of the actively growing microbial layer from a Shark Bay stromatolite (Burns et al., 2004).

Cyanobacterial proportion (26.4% of reads) in freshwater mats from Giblin River was extensive, with the main phylotypes most similar to cyanobacteria from soil crusts, freshwater lakes and biofilms (Proemse et al., 2017). Representatives of the *Scytonemataceae* (including *Chakia*, *Scytonema* and *Ewamiania*) were the most active heterocytous cyanobacteria in Giblin River microbial mats based on their high relative abundance of 16S rRNA transcripts, which was also supported by the predominance of hexose HG_30_ triols that have been reported exclusively from members of the genus *Scytonema* so far (Bauersachs et al., 2019; Gambacorta et al., 1999). *Scytonema* mats have also been identified in a karstic freshwater lake on the Yucatan Peninsula, Mexico (Gischler et al., 2011). These mats show morphological similarities to the green mats sampled from Giblin River, forming firmly lithified hemispheroidal structures, densely covered by a cyanobacterial biofilm, which was internally permeated throughout by calcified sheaths.

### Physiological activities of heterocytous cyanobacteria in the microbial mats

Heterocytous cyanobacteria made up relatively small proportions of the overall active microbial communities in (hyper)saline mats (3.1%–9.5%) but were found to contribute intensively to gene transcripts associated with nitrogen fixation (15.7%–44.9%) and biofilm production (7.1%–34.9%). Cyanobacteria occurring in the upper aerobic layer of the microbial mats (2–3 mm) are considered as the most important primary producers of all phototrophic mat types (Stal, 1995). This study found high relative abundances of photosynthetic and carbon fixation gene transcripts from Chroococcales in the saline mats, with smaller contributions from Oscillatoriales and Nostocales. However, Nostocales made greater contributions to photosynthetic carbon fixation pathways in freshwater green mats. Chroococcales have been shown to make the largest contribution to photosynthesis‐related gene transcripts in coastal microbial mats (Hörnlein et al., 2018), whereas in freshwater microbial mats from Antarctica, Nostocales were reported to be the major primary producers (Almela et al., 2019). In agreement with these findings, our study demonstrates that based on combined 16S rRNA and lipid biomarker data, heterocytous cyanobacteria are likely the main primary producers in freshwater microbial mats of Giblin River.

Heterocytous cyanobacteria were also found to be prominent biofilm producers in a majority of microbial mat types studied. *Anabaena* spp. and *Nostoc* spp. are known to be major producers of EPS (Cruz et al., 2020; De Philippis & Vincenzini, 1998; Singh et al., 2016). EPS play an important role in providing anchorage to substrate(s), to allow protection against desiccation, predation, masking of antibody recognition, and prevention of lysis by viruses and other bacteria (P. Li et al., 2001). Furthermore, cyanobacteria yield EPS as a direct response to selective pressures from their environment, such as pH and salinity (Rossi & De Philippis, 2015). Therefore, the selective pressures faced by heterocytous cyanobacteria occurring in the microbial mats investigated here indicate that they are likely playing an essential role in the formation of biofilms and ongoing protection of the overall microbial community.

A previous study assessing *nif*H transcripts indicative of active diazotrophic communities in coastal‐microbial mats found that Oscillatoriales were dominant under freshwater conditions, whereas Chroococcales were abundant under marine conditions (Severin et al., 2012). Under elevated salinities, both orders as well as Nostocales were found, but the overall contribution of cyanobacteria to the *nif*H transcript libraries was noticeably lower at increasing salinity. Another study detecting *nif*H transcripts found that communities dominated by heterocytous cyanobacteria exhibited light‐independent nitrogen fixation at total salinity (≤60); whereas, communities dominated by non‐heterocytous cyanobacteria exhibited nitrogen fixation at total salinity ≤100 (Namsaraev et al., 2018). The use of metatranscriptomics has enabled for a larger group of *nif* transcripts to be observed, with many *nif* transcripts (i.e. *nif*B or *nif*X) being highly transcribed by Nostocales in the mats. This puts into question the reliability of the *nif*H transcript as an indicator for overall diazotrophic activity. A majority of the *nif* genes observed in this study are part of a large gene cluster including *nif*B‐*fdx*N‐*nif*S‐*nif*U‐*nif*H‐*nif*D‐*nif*K‐*nif*E‐*nif*N‐*nif*X found within the *Anabaena* genome (Haselkorn, 1986). Furthermore, nitrogenase gene expression in *Anabaena variabilis* indicated that *nif*B appeared to be the primary promoter for the entire *nif* cluster and that structural genes *nif*HDK were the most abundant transcripts (Pratte & Thiel, 2014). However, their abundance was not controlled by an independent *nif*H promoter. Due to the abundance of the *nif*B transcript in nostocalean transcriptomes from Shark Bay microbial mats, we conclude that the *nif*B transcript may pose a more reliable indicator of diazotrophic activity of heterocytous cyanobacteria occurring in enhanced saline environments.

Our study also demonstrates that Nostocales and/or Chroococcales transcribed the greatest extent of nitrogen‐fixing genes in metahaline and hypersaline environments with similar amounts of these genes being transcribed during the day and night. This suggests that representatives of both orders play a similar role in nitrogen acquisition within saline mats from Shark Bay. In the freshwater environment of Giblin River, heterocytous cyanobacteria were found to be the only diazotrophs in the green mats, whereas Rhizobiales (α‐Proteobacteria) made major contributions in nitrogen‐fixing transcripts (57.1%) in the YM. In addition, the detection of nitrogen‐fixing gene transcription was higher in the saline mats when compared to the freshwater mats, suggesting that the marine environment of Shark Bay is more depleted in available nitrogen than that of Giblin River.

### Sources and environmental controls on HG distributions

HGs are exclusively found in the heterocyte cell envelope and as such represent unique biological markers for the presence and activity of heterocytous cyanobacteria and the process of N_2_ fixation (Bauersachs, Compaoré, et al., 2009; Gambacorta et al., 1999). PCA of molecular and HG data showed that the freshwater and marine microbial mats mainly grouped together based on sampling location but not based on the type of mat or time of sampling. This suggests that the environmental conditions of the habitat mainly control the community composition of heterocytous cyanobacteria. Nilemah (hypersaline) and Garden Point mats (metahaline) grouped together based on the taxonomic data but were separated based on their HG distributions, suggesting salinity could be controlling HG synthesis in cyanobacteria in intertidal/subtidal zones. Hexose HG_28_ diol was highly abundant in all metahaline to hypersaline microbial mats and found to correlate with mats containing high proportions of Nostocales. This component has previously been reported to be abundant in cultures of *Anabaena* spp. and *Nostoc* spp. (Bauersachs, Compaoré, et al., 2009), with both genera belonging to the most dominant heterocytous cyanobacteria in the microbial mats investigated here. RRBS and Nilemah mats were rich in HGs with pentose headgroup attached to diols and triols of 30–32 carbon atoms. These components have been reported exclusively from the cyanobacterial endosymbiont *Richelia intracellularis* found in marine diatoms of the genus *Hemiaulus* (Schouten et al., 2013). Although *Richelia* sp. was indeed confirmed in a microbial mat from RRBS based on 16S rRNA gene sequencing, it was below detection in other RRBS and Nilemah mats and other cyanobacterial sources might have to be considered for this component. The freshwater microbial mats from Giblin River primarily contained HGs with hexose headgroup that are highly characteristic for freshwater cyanobacteria, but which are also common in coastal microbial mats (Bauersachs et al., 2011; Bühring et al., 2014). Molecular and HG data both indicate that mats from these locations were taxonomically similar dominated by members of the Scytonemataceae (including *Scytonema* spp. and *Ewamiania* spp.) and Rivulariaceae (*Calothrix* spp.), in agreement with high abundances of hexose HG_30_ triols (Bauersachs et al., 2019; Gambacorta et al., 1999) and hexose HG_28_ triols (Bauersachs et al., 2019; Bauersachs, Compaoré, et al., 2009; Gambacorta et al., 1999; Wörmer et al., 2012) in these mats. Linke Lake had the highest salinity (>80) and contained the lowest diversity of heterocytous cyanobacteria, mainly comprising true‐branching *Fischerella*, *Chlorogloeopsis* and *Stigonema* with smaller amounts of non‐branching *Nostoc*, *Tolypothrix* and *Scytonema* (Figure 5). This is largely in line with the high abundances of hexose HG_26_ diols (Bauersachs, Compaoré, et al., 2009; Wörmer et al., 2012), hexose HG_28_ triols (Bauersachs et al., 2019; Wörmer et al., 2012) and hexose HG_30_ triols (Bauersachs et al., 2019; Gambacorta et al., 1999) in the microbial mat collected from Linke Lake (Figure 7). Me‐hexose HG_28_ triol was abundantly present in all microbial mat samples, regardless of salinity (Figure 7). This HG has been identified only in an isolate of the free‐living marine cyanobacterium *Calothrix* sp. to date and suggested as a biological marker for exclusively marine conditions (Bale et al., 2018). Its ubiquitous presence in freshwater to hypersaline microbial mats, however, suggests that this component likely has multiple biological sources within the cyanobacterial realm. Future research should focus on establishing HG distribution patterns in cyanobacterial cultures to improve their value for profiling cyanobacterial communities in coastal‐microbial microbial mats.

## CONCLUSION

Taxonomic and functional analysis based on metatranscriptomics revealed that the diversity and physiological activity of heterocytous cyanobacteria in coastal‐marine microbials mats is substantially greater than previously considered. Analysis of microbial mats occurring in a diverse range of environmental settings also revealed a high diversity of HGs, which further corroborates with the molecular‐inferred taxonomic diversity indicating several cyanobacterial sources. The combined metatranscriptomic and lipidomic approach applied in this study demonstrates the potential to study the diversity and activity of heterocytous cyanobacteria in microbial mats developing under various salinity regimes in a yet unprecedented detail.

## AUTHOR CONTRIBUTIONS

Field sampling of microbial mats was conducted by Matthew A. Campbell, Kliti Grice and Marco J. L. Coolen in Shark Bay, and Matthew A. Campbell, Bernadette C. Proemse and Rolan S. Eberhard in Giblin River. Laboratory sample processing was done by Matthew A. Campbell. Lipid analysis was conducted by Thorsten Bauersachs and Lorenz Schwark. Statistical and computational analyses were performed by Matthew A. Campbell. The manuscript was reviewed and edited by all authors.

## CONFLICT OF INTEREST

The authors declare no competing interests.

## Supporting information


**Appendix S1:** Supporting Information.

## Data Availability

Pre‐assembled and assembled reads have been deposited on osf.io/e94yg under the project “Shark Bay Transcriptomics”.
