# Supplementary material for: Salinity‐driven ecology and diversity changes of heterocytous cyanobacteria in Australian freshwater and coastal‐marine microbial mats
Source: Environ Microbiol. 2022 Oct 10;24(12):6493–509. doi: 10.1111/1462-2920.16225 (PMC10092834; doi:10.1111/1462-2920.16225)
Supplement: Supplementary file 1 — Appendix S1: Supporting Information. [file EMI-24-6493-s001.docx]

Supplementary Information for

**Salinity-driven ecology and diversity changes of heterocytous cyanobacteria in Australian freshwater and coastal-marine microbial mats**

Matthew A. Campbell, Thorsten Bauersachs, Lorenz Schwark, Bernadette C. Proemse, Rolan S. Eberhard, Marco J.L. Coolen, Kliti Grice

Email: matthew.campbell@curtin.edu.au

This file includes:

Supplementary Figures 1 to 5

Supplementary Tables 1 to 10


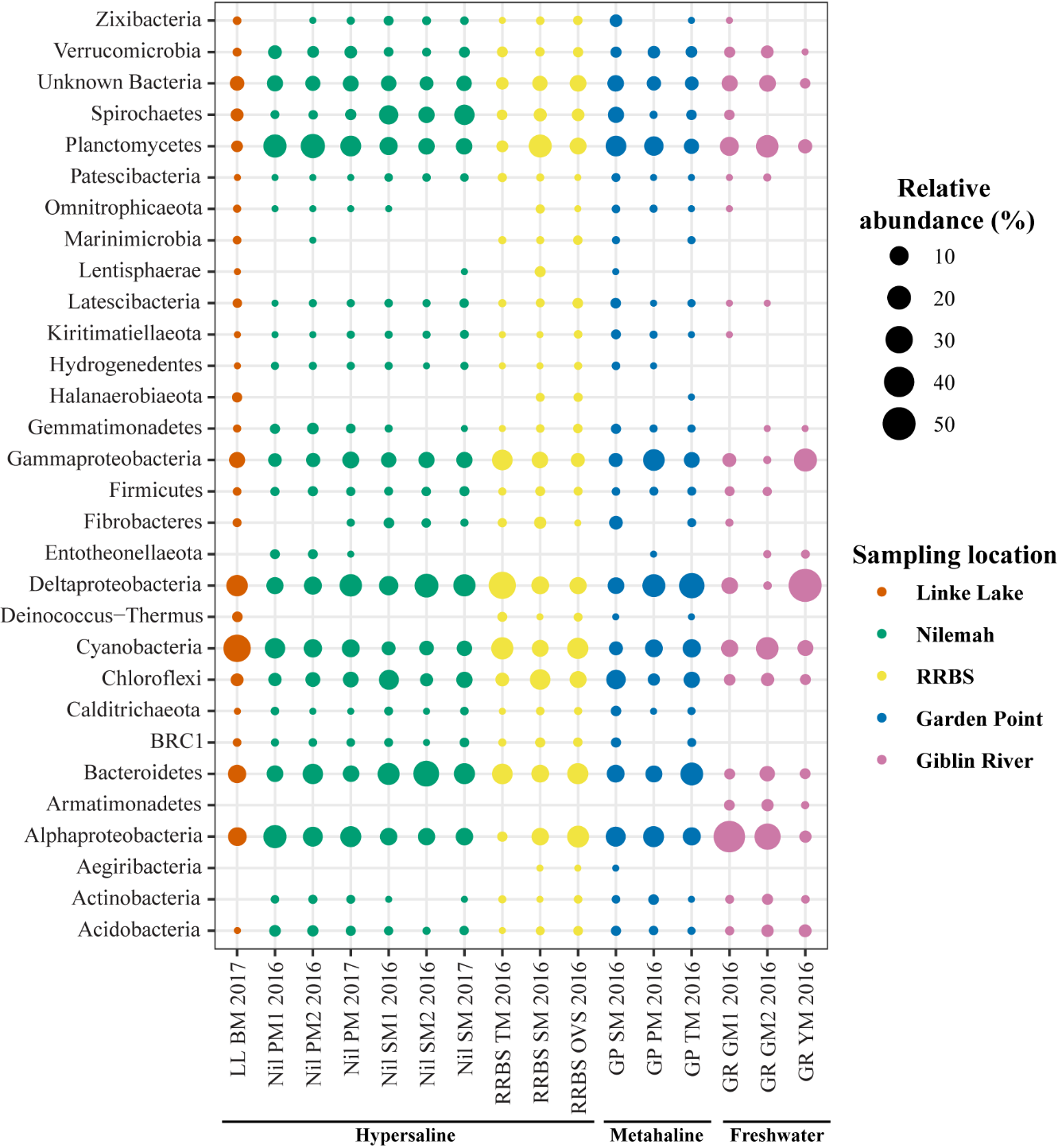
***Supplementary Fig 1*** Dot plot displaying the composition and abundance of the top 30 most abundant taxa (phylum/class) in microbial mats from Shark Bay, Western Australia, and Tasmania. Linke Lake (LL), Nilemah (Nil), RRBS and Garden Point (GP), Giblin River (GR). BM = birrida mat (gelatinous mat), PM = pustular mat, SM = smooth mat, TM = tufted mat, OVS= ooze over sand, GM = green mat, and YM = yellow mat.

***
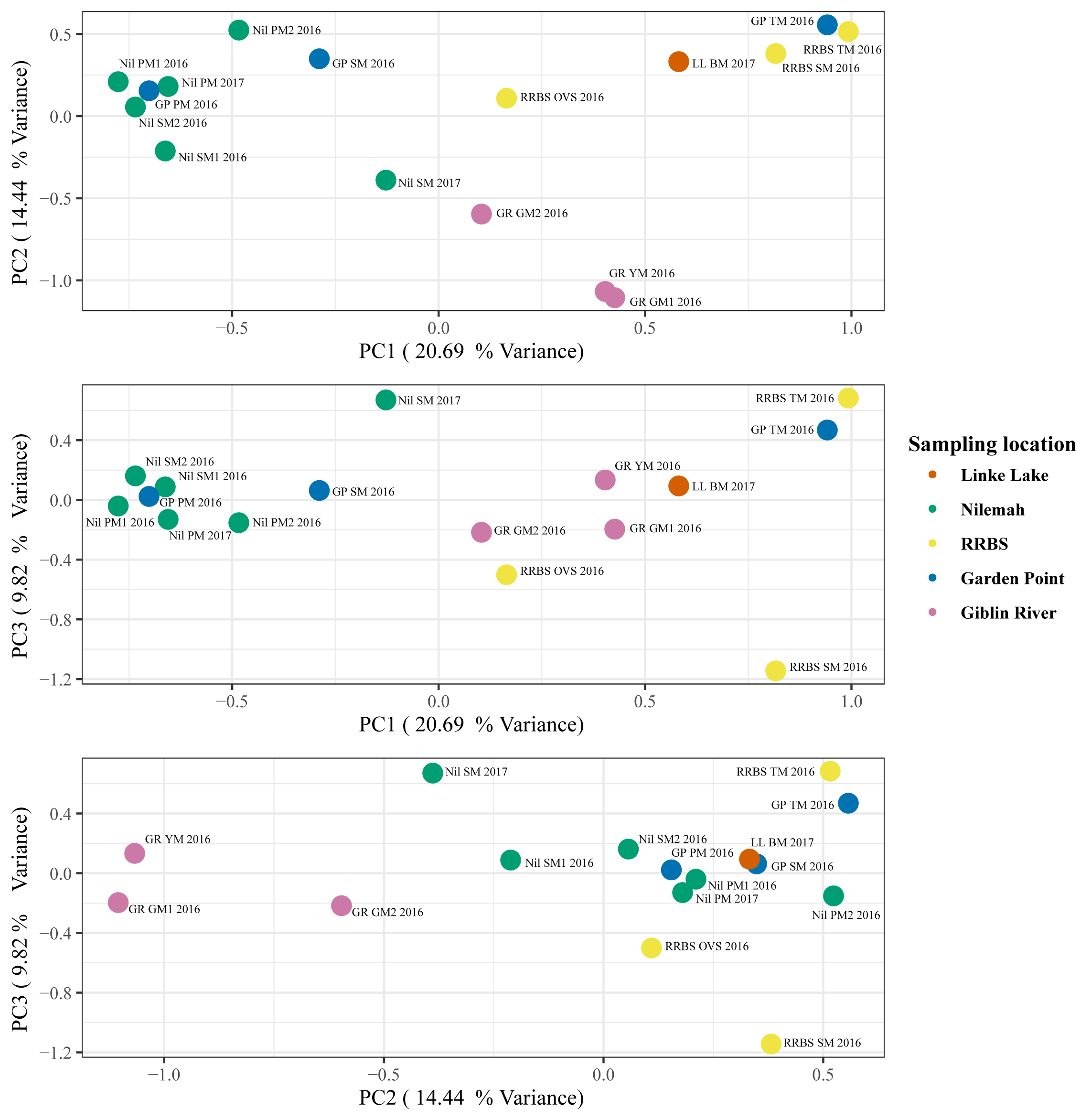
***

***Supplementary Fig 2*** PCA plot constructed from similarity matrices utilising 16S rRNA gene recruitment of non-branching (Nostocales) and true-branching (Stigonematales) heterocytous cyanobacteria in microbial mats from Shark Bay, Western Australia, and Tasmania. Linke Lake (LL), Nilemah (Nil), RRBS and Garden Point (GP), and Giblin River (GR). BM = birrida mat (gelatinous mat), PM = pustular mat, SM = smooth mat, TM = tufted mat, OVS= ooze over sand, GM = green mat, and YM = yellow mat.


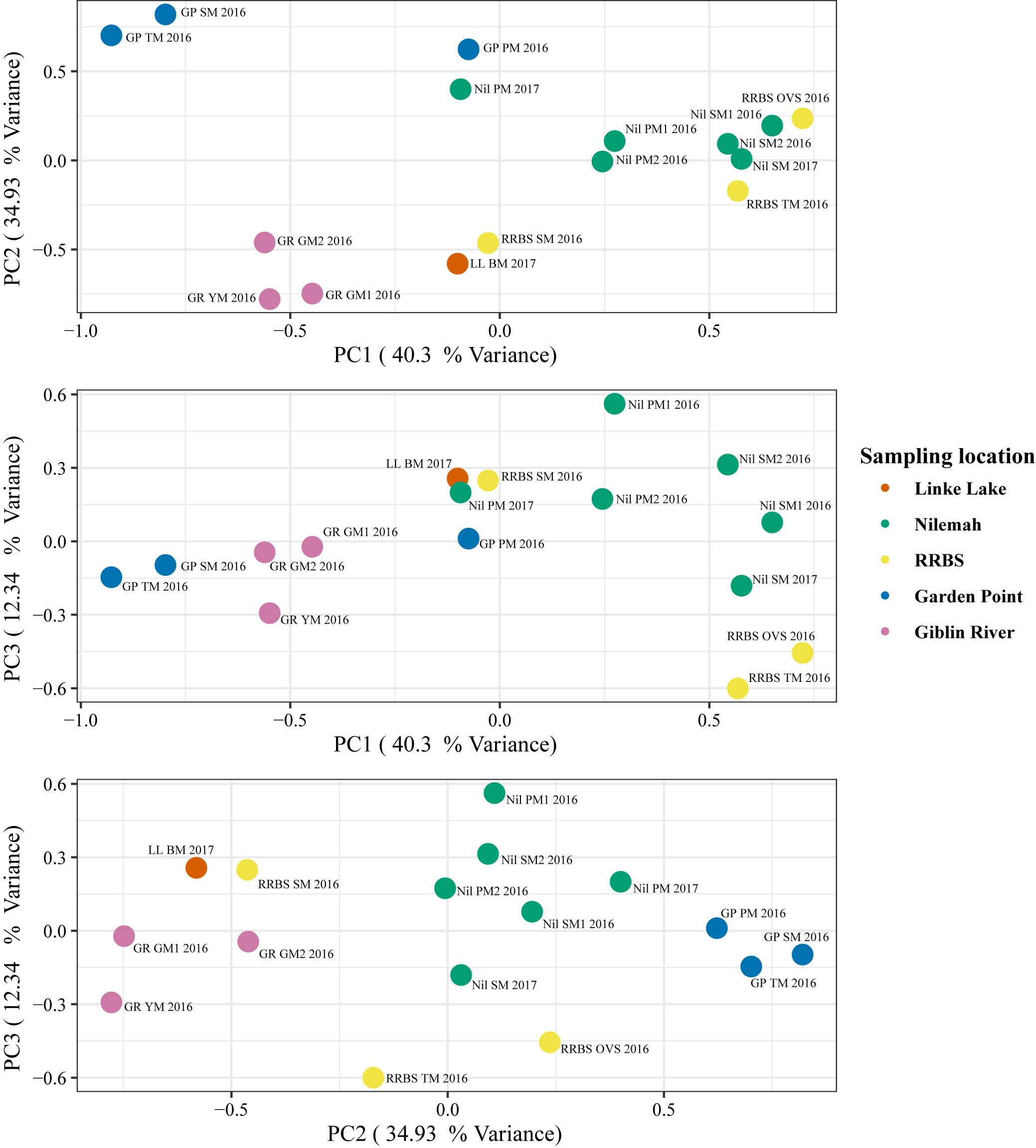


***Supplementary Fig 3*** PCA plots constructed from similarity matrices utilising fractional abundances of heterocyte glycolipids (HGs) within microbial mats from Shark Bay, Western Australia, and Tasmania. Linke Lake (LL), Nilemah (Nil), RRBS and Garden Point (GP), and Giblin River (GR). BM = birrida mat (gelatinous mat), PM = pustular mat, SM = smooth mat, TM = tufted mat, OVS= ooze over sand, GM = green mat, and YM = yellow mat.


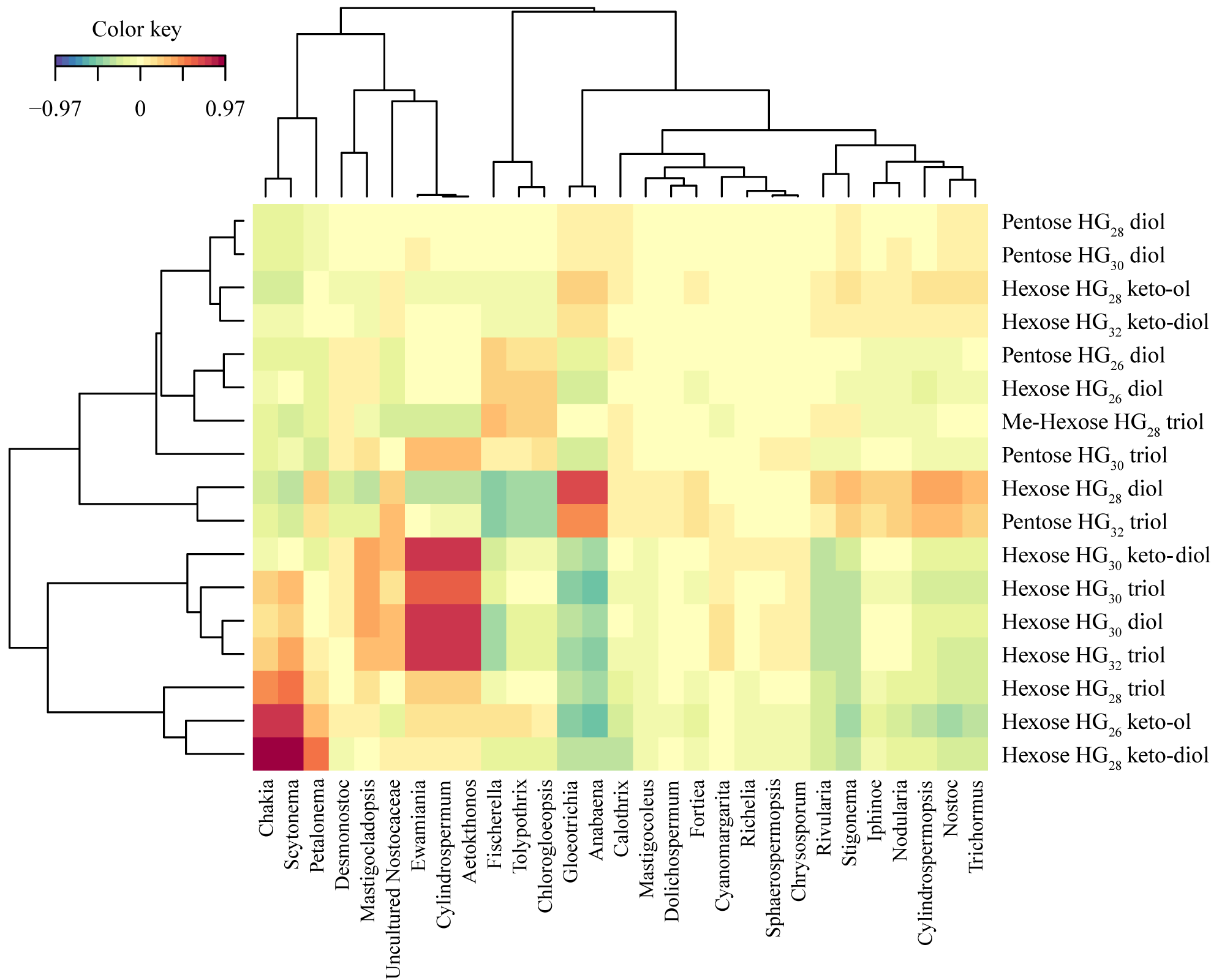


***Supplementary Fig 4*** Heatmap analysis performed by using regularized canonical correlations analysis showing the relation between taxonomic and lipidomic datasets. Correlation strengths are indicated by the colour key.

***
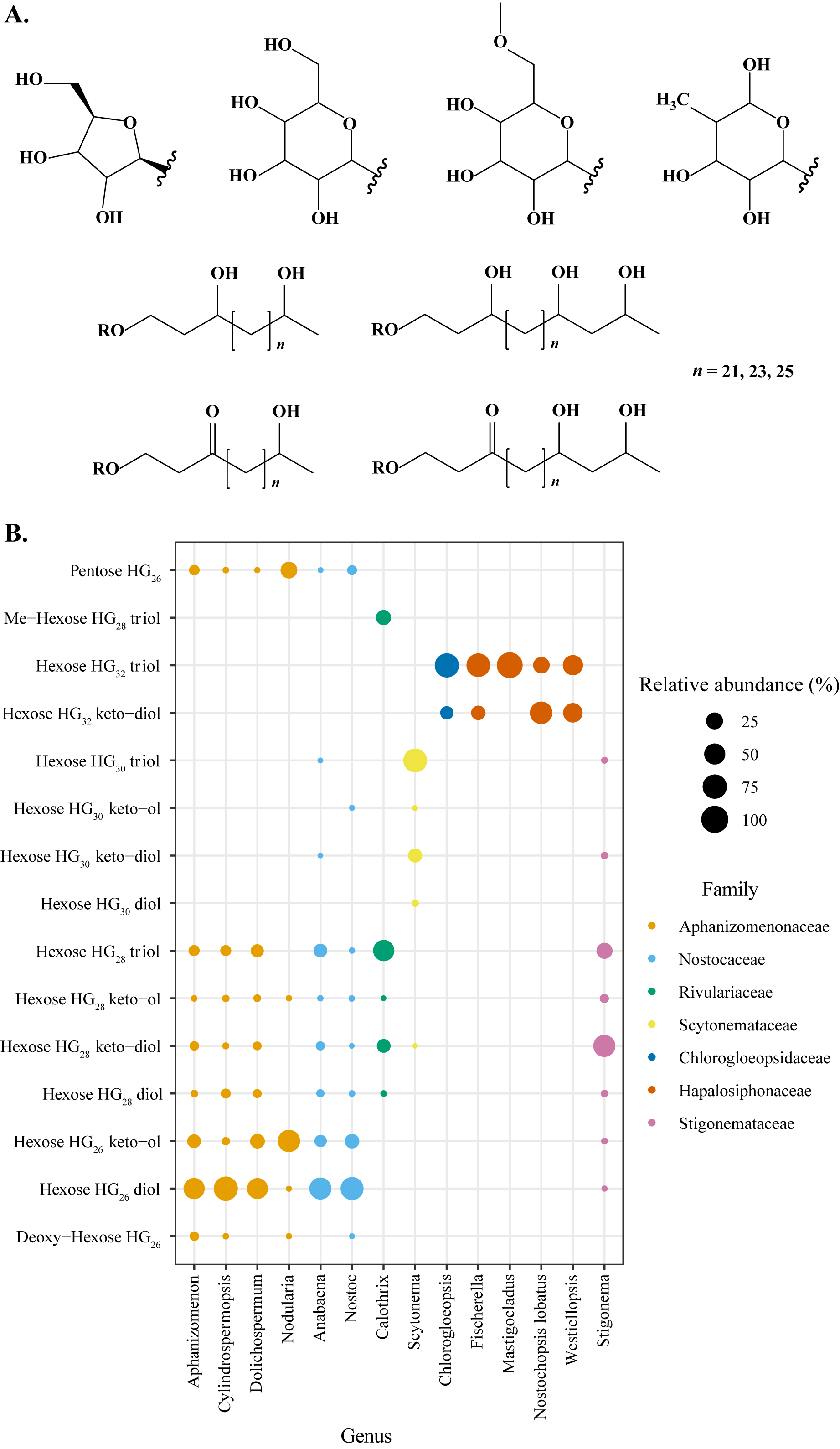
***

***Supplementary Fig 5*** HG structures and their distribution in cultured cyanobacteria. A. Sugar head groups (pentose-, hexose-, methyl-hexose and deoxy-hexose), functional moieties (diol, triol, keto-ol, and keto-diol) and chain lengths. B. Relative abundance of HGs described in cyanobacterial isolates (e.g. Gambacorta et al., 1999; Bauersachs et al., 2009, 2013, 2014, 2019; Wörmer et al., 2012).

***Supplementary Table 1*** Summary of the mat types collected with locations, sampling dates and times, field measurements of salinity, pH and water temperature, geomorphic settings and morphological features.

| **Sample ID** | **Location** | **Mat type** | **Sampling date** | **Sampling time** | **Salinity** | **pH** | **Water temperature (°C)** | **Geomorphic setting** | **Morphological feature** |
| --- | --- | --- | --- | --- | --- | --- | --- | --- | --- |
| Nil PM1 2016 | Nilemah | Pustular mat | 5/07/2016 | 12:40 PM | 67 | 7.94 | 13.2 | intertidal terrace front | microbialite ridge crest |
| Nil PM2 2016 | Nilemah | Pustular mat | 5/07/2016 | 12:10 PM | 66 | 8.06 | 13.2 |  | continuous mat sheet |
| Nil PM 2017 | Nilemah | Pustular mat | 10/04/2017 | 8:30 AM | 70 | 7.51 | 18.0 |  | microbialite ridge crest |
| Nil SM1 2016 | Nilemah | Smooth mat | 5/07/2016 | 12:00 PM | 66 | 7.99 | 13.2 |  |  |
| Nil SM2 2016 | Nilemah | Smooth mat | 5/07/2016 | 12:30 PM | 67 | 7.93 | 13.2 |  | swale between microbialite ridges |
| Nil SM 2017 | Nilemah | Smooth mat | 10/04/2017 | 8:45 PM | 70 | 7.51 | 18.0 |  |  |
| LL BM 2017 | Linke Lake | Gelatinous mat | 11/04/2017 | 3:30 PM | >80 | 7.75 | 15.2 | birrida - moat-like depression | evaporative pond |
| GP PM 2016 | Garden Point | Pustular mat | 6/07/2016 | 12:00 PM | 51 | 7.85 | 14.0 | intertidal | mat sheet |
| GP SM 2016 | Garden Point | Smooth mat | 6/07/2016 | 11:00 AM | 51 | 7.85 | 14.0 | subtidal | sand sheet |
| GP TM 2016 | Garden Point | Tufted mat | 6/07/2016 | 11:30 AM | 51 | 7.85 | 14.0 | intertidal | continuous mat sheet |
| RRBS TM 2016 | RRBS | Tufted mat | 7/07/2016 | 12:12 PM | 69 | 7.60 | 15.0 | intertidal terrace | mat sheet |
| RRBS SM 2016 | RRBS | Smooth mat | 7/07/2016 | 1:25 PM | 68 | 7.17 | 15.0 | within outwash channel delta sand sheet | mat sheet patch |
| RRBS OVS 2016 | RRBS | Transitioning mat | 7/07/2016 | 1:35 PM | 66 | 7.21 | - |  | new sand sheet |
| GR GM1 2016 | Giblin River | Green mat | 18/8/2016 | 10:45 AM | 0.24 | 7.68 | 14.3 | karstic wetland | calcareous mud and tufa |
| GR YM 2016 | Giblin River | Yellow mat | 18/8/2016 | 1:30 AM | 0.16 | 7.08 | 16.1 |  | calcareous mud and tufa |
| GR GM2 2016 | Giblin River | Green mat | 18/8/2016 | 3:15 PM | 0.06 | 7.96 | 13.0 |  | creek bank |

***Supplementary Table 2*** Illumina HiSeq 2500 pair-end sequencing read output with percentage summaries of trimmed and aligned sequences, number of assembled transcripts and number of annotations for Phyloflash and eggNOG-mapper.

|  | **Percentage of trimmed and aligned sequences** | | | | | | **# annotations** | |
| --- | --- | --- | --- | --- | --- | --- | --- | --- |
|  |  |  |  |  |  |  | **PhyloFlash (SSU rRNA SILVA)** | **eggNOG-mapper** |
| **Sample Name** | **Paired Reads** | **Quality trimming** | **Remaining reads** | **aligned 0 times** | **aligned >1 times** | **# transcripts assembled** | **Total** | **Total** |
| Nil PM1 2016 | 1.10E+07 | 8.87 | 91.13 | 7.10 | 92.90 | 26890 | 1474649 | 102200 |
| Nil PM2 2016 | 9066195 | 7.71 | 92.29 | 7.57 | 92.43 | 9393 | 1652757 | 42492 |
| Nil PM 2017 | 9184291 | 9.86 | 90.14 | 9.39 | 90.61 | 29423 | 1827324 | 121125 |
| Nil SM1 2016 | 9621836 | 8.49 | 91.51 | 8.41 | 91.59 | 25348 | 1707460 | 121088 |
| Nil SM2 2016 | 9771353 | 9.38 | 90.62 | 15.57 | 84.43 | 63494 | 905558 | 137750 |
| Nil SM 2017 | 9214600 | 8.74 | 91.26 | 9.47 | 90.53 | 24822 | 1293097 | 151598 |
| LL BM 2017 | 9810574 | 8.85 | 91.15 | 6.59 | 93.41 | 16412 | 1453025 | 64193 |
| GP PM 2016 | 8509997 | 8.39 | 91.61 | 10.10 | 89.90 | 27442 | 1850380 | 92066 |
| GP SM 2016 | 8966989 | 8.63 | 91.37 | 8.52 | 91.48 | 14554 | 1206356 | 123465 |
| GP TM 2016 | 8890215 | 14.87 | 85.13 | 58.27 | 41.73 | 14284 | 751314 | 238791 |
| RRBS TM 2016 | 9189088 | 8.73 | 91.27 | 9.55 | 90.45 | 28538 | 778082 | 114382 |
| RRBS SM 2016 | 8084891 | 16.74 | 83.26 | 31.44 | 68.56 | 48288 | 1040348 | 217075 |
| RRBS OVS 2016 | 9975457 | 8.47 | 91.53 | 7.12 | 92.88 | 19737 | 1683164 | 92373 |
| GR GM1 2016 | 8674558 | 9.39 | 90.61 | 7.19 | 92.81 | 8192 | 2322053 | 49295 |
| GR YM 2016 | 9232190 | 10.90 | 89.10 | 95.29 | 4.71 | 447678 | 45821 | 2013150 |
| GR GM2 2016 | 8732656 | 9.14 | 90.86 | 4.66 | 95.34 | 13725 | 1595651 | 42926 |

***Supplementary Table 3*** Relative abundance (%) of cyanobacterial 16S rRNA transcripts from microbial mats occurring in hypersaline, metahaline and freshwater environments.

***Supplementary Table 4*** Relative abundance (%) of cyanobacterial mRNA transcripts from microbial mats occurring in hypersaline, metahaline and freshwater environments.

***Supplementary Table 5*** Relative transcript abundances (%) from taxa (domain/phylum and order levels) transcribing genes involved in photosynthesis in microbial mats occurring in hypersaline, metahaline and freshwater environments.

***Supplementary Table 6*** Relative transcript abundances (%) from taxa (phylum and order levels) transcribing genes involved in carbon fixation in microbial mats occurring in hypersaline, metahaline and freshwater environments.

***Supplementary Table 7*** Relative transcript abundances (%) from taxa (phylum and order levels) transcribing genes involved in exopolysaccharides (EPS) and capsular polysaccharides (biofilm formation) in microbial mats occurring in hypersaline, metahaline and freshwater environments.

***Supplementary Table 8*** Relative transcript abundances (%) from taxa (phylum and order levels) transcribing genes involved in nitrogen fixation in microbial mats occurring in hypersaline, metahaline and freshwater environments.

***Supplementary Table 9*** Multilevel pattern analysis of nostocalean cyanobacteria.

| **Genera** | **Nilemah** | **Garden Point** | **RRBS** | **Linke Lake** | **Giblin River** | **Index** | **Stat** | **P.Value** |
| --- | --- | --- | --- | --- | --- | --- | --- | --- |
| ***Gloeotrichia*** | 1 | 1 | 0 | 0 | 0 | 6 | 0.942 | 0.01 |
| ***Anabaena*** | 1 | 1 | 0 | 0 | 0 | 6 | 0.992 | 0.003 |
| ***Rivularia*** | 1 | 1 | 0 | 0 | 0 | 6 | 0.914 | 0.065 |
| ***Chakia*** | 0 | 0 | 0 | 0 | 1 | 5 | 1.000 | 0.011 |
| ***Petalonema*** | 1 | 0 | 0 | 0 | 1 | 9 | 0.891 | 0.062 |

***Supplementary Table 10*** Multilevel pattern analysis of heterocyte glycolipids (HGs).

| **HG** | **Nilemah** | **Garden Point** | **RRBS** | **Linke Lake** | **Giblin River** | **Index** | **Stat** | **P.Value** |
| --- | --- | --- | --- | --- | --- | --- | --- | --- |
| **Pentose HG_30_ diol** | 1 | 1 | 1 | 0 | 0 | 16 | 0.968 | 0.047 |
| **Pentose HG_30_ triol** | 0 | 0 | 1 | 1 | 1 | 25 | 0.925 | 0.063 |
| **Pentose HG_32_ triol** | 0 | 0 | 1 | 1 | 1 | 25 | 0.918 | 0.069 |
| **Pentose HG_28_ diol** | 1 | 1 | 1 | 0 | 1 | 27 | 1.000 | 0.070 |
| **Hexose HG_26_ keto-ol** | 1 | 0 | 1 | 1 | 1 | 15 | 0.991 | 0.151 |
| **Hexose HG_30_ diol** | 0 | 0 | 0 | 0 | 1 | 5 | 1.000 | 0.009 |
| **Hexose HG_32_ keto-diol** | 1 | 0 | 0 | 0 | 1 | 5 | 0.666 | 0.563 |
| **Hexose HG_32_ triol** | 0 | 0 | 0 | 0 | 1 | 5 | 1.000 | 0.009 |

**REFERENCES**

Bale N. J., Hopmans E. C., Dorhout D., Stal L. J., Grego M., van Bleijswijk J., Sinninghe Damsté J. S. and Schouten S. (2018) A novel heterocyst glycolipid detected in a pelagic N_2_-fixing cyanobacterium of the genus Calothrix. *Org. Geochem.* **123**, 44–47.

Bale N. J., Hopmans E. C., Zell C., Sobrinho R. L., Kim J. H., Sinninghe Damsté J. S., Villareal T. A. and Schouten S. (2015) Long chain glycolipids with pentose head groups as biomarkers for marine endosymbiotic heterocystous cyanobacteria. *Org. Geochem.* **81**, 1–7.

Bauersachs T., Compaoré J., Hopmans E. C., Stal L. J., Schouten S. and Sinninghe Damsté J. S. (2009) Distribution of heterocyst glycolipids in cyanobacteria. *Phytochemistry* **70**, 2034–2039.

Bauersachs T., Miller S. R., Gugger M., Mudimu O., Friedl T. and Schwark L. (2019) Heterocyte glycolipids indicate polyphyly of stigonematalean cyanobacteria. *Phytochemistry* **166**, 112059.

Bauersachs T., Miller S. R., van der Meer M. T. J., Hopmans E. C., Schouten S. and Sinninghe Damsté J. S. (2013) Distribution of long chain heterocyst glycolipids in cultures of the thermophilic cyanobacterium *Mastigocladus laminosus* and a hot spring microbial mat. *Org. Geochem.* **56**, 19–24.

Bauersachs T., Mudimu O., Schulz R. and Schwark L. (2014) Distribution of long chain heterocyst glycolipids in N_2_-fixing cyanobacteria of the order Stigonematales. *Phytochemistry* **98**.

Gambacorta A., Trincone A., Soriente A. and Sodano G. (1999) Chemistry of glycolipids from the heterocysts of nitrogen-fixing cyanobacteria. *Curr. Top. Phytochem.* **2**, 145–150.

Wörmer L., Cirés S., Velázquez D., Quesada A. and Hinrichs K.-U. (2012) Cyanobacterial heterocyst glycolipids in cultures and environmental samples: Diversity and biomarker potential. *Limnol. Oceanogr.* **57**, 1775–1788.
